# Supplementary material for: Automated reporting of cervical biopsies using artificial intelligence
Source: PLOS Digit Health. 2024 Apr 22;3(4):e0000381. doi: 10.1371/journal.pdig.0000381 (PMC11034655; doi:10.1371/journal.pdig.0000381)
Supplement: Attachment — Submitted filename: Response to Reviewers.docx [file pdig.0000381.s002.docx]

Q1. Please indicate out of the 2539 whole slide images (WSIs), the number of images that were obtained from Glasgow Royal Infirmary (NG), Southern General Hospital (SG), Royal Alexandria Hospital (RAH) and Queen Elizabeth University Hospital (QEUH).

The number of tissue blocks obtained from each of the above sites were: 829 from QEUH, 729 from NG, 647 from SG and 334 from RAH.

Note: Addressed in lines 91-93.

Q2. Please indicate the imaging parameters of the Philips UFS Scanner including resolution, FOV; and the Magnification of the equipment.

All slides were then scanned at QEUH and saved as WSI. The WSI are hundreds of thousands of pixels in height and width at the highest magnification and are too large to read into memory. Dedicated WSI formats allow access to either small parts of the image at the highest magnification or the whole image at lower magnifications. For this study, the slides were scanned using a Phillips Ultra Fast Scanner (UFS) with resolution equivalent to 40x or more specifically 0.25 microns/pixel, and stored in the isyntax file format. The most detailed view in the WSI is level 0, or 40x magnification where the length of a side of 1 pixel in the image is 0.25µm. Higher levels represent lower magnifications in a pyramid where each level is a power of 2 smaller than the previous. For example at level 5, one pixel represents a square patch at level 0 with a length of 2^5^= 32 pixels per side, or an area of 32 × 32 = 1024 pixels in total.

FOV is Field of view is not relevant as it depends on the resolution of the monitor.

Note: Addressed in lines 100-110

Q3. On line 140, you mention that two of the labs (labs 6 & 8) and 10% of WSIs from other labs which were randomly. Please indicate which are the labs 6 & 8 mentioned in the manuscript.

New tissue sections were cut from the tissue blocks at one of two different thicknesses (3 microns or 4 microns) and then stained with one of four different H&E protocols (routine H\&E, muscle biopsy protocol, neuro protocol and paeds protocol). Together, these combinations gave eight different labs maximising WSI variance and thereby decrease the likelihood of overfit to any one lab (combination of tissue processing, cutting and staining protocol.

Note: Addressed in lines 94-99

Q4. Justify why you selected 33% of the training slides as your test train split. Was increasing or reducing this affecting your results in any way (Line 142). However, your Table 1 indicates otherwise. Please clearly explain your test train split.

We received a total of 2539 whole slide images (WSIs), with only one slide per patient, in iSyntax format, an Excel file containing metadata, categories, and subcategories and an annotation file per WSI in JSON format at the end of the annotation process. These WSIs were split into training, validation and test sets as shown in Table 1. The split percentages were calculated based on the case labels associated with the samples recorded in the system and the numbers per each set were agreed on by all the team members and the pathologists. All slides from two of the labs and 10% randomly selected slides from the other six labs were set aside as test set and never used in the training and validation process. The remaining 90\% of the slides, from the 6 other labs were used as training and validation sets. Two third of these slides were randomly selected and used as validation set. To retain the the same proportion of classes in the train and test sets that are found in the entire original dataset, dataset was split using a stratified fashion balanced over categories, subcategories and staining labs for training, validation and test sets. During the annotation process these labels were doubled checked and in approximately 5% of the cases the final label associated with the scanned slide was different. This could be because the new slice taken from the sample did not show the same pathology as the original or that the original label was incorrectly recorded. The corrected labels post annotation were the labels that were used for training and testing. This means the final numbers of slides of each type may not match the original percentages described above.

Note: This is addressed in section 2.2 lines 152-171

Q5. Avoid putting tables in between a sentence. Place Table 2 after paragraph 297 to improve readability.

All tables and figures are placed properly at the end of the paragraphs.

Q6. XGBoost classifier is an excellent algorithm that has been used by several other authors. Can you please compare your results (probably some metrics of the confusion matrix) with some of the state of art available algorithms with a special focus on the XGBoost classifier variations proposed by different authors.

Most of the available papers on cervical cancer detection are on CT/MRI/PET-CT, pop smear and cytology images and they perform different type of segmentation and classification tasks. A recent review of artificial intelligence in gynecological cancers [29] found thirty-four studies on cervical cancer, of which 18 used imaging data and 16 used value-based data. Three studies used MRI images to predict the stage of cervical cancer. Fifteen studies used CT/MRI/PET-CT images to predict recurrence and metastasis. Ten studies used clinical parameters to predict the diagnosis. Six studies used clinical parameters to predict therapeutic courses. The dataset used in our paper has not been used before and the task and the categories we perform classification on, are different. Despite these facts, we try to compare the performance of our algorithm with some of the related state of the art papers.

The proposed algorithm in [12] is a progressive lesion cell recognition method combining low-and high-resolution WSIs to recommend lesion cells and a recurrent neural network-based (RNN) WSI classification model to evaluate the lesion degree of cervical WSIs. On independent test sets of 1,170 patient-wise WSIs, this proposed algorithm has reached 95.1% Sensitivity for classifying slides. In [30] a predictive model consists of two main phases is proposed. A Mask R-CNN model segments and classify cells in all ThinPrep cytologic test (TCT) images from patients into three classes and then a machine-learning model is used to classify patients into two classes (normal vs. abnormal), using the combination of cell classification from the best models constructed (T1, A3) and clinic diagnosis results. Table 9 shows comparison of the performance of our algorithm with some of the state of the art algorithms for cervical cancer classification considering the fact that the type of images used in these papers are different from ours.

| Paper | Dataset | Algorithm | Test Slides | Sensitivity |
| --- | --- | --- | --- | --- |
| Our paper | Hematoxylin and Eosin Stained (H & E) Whole Slide Images | GoogLeNet+ XGBoost | 801 | 93.40% |
| [12] | Cytology Whole Slide Images | RNN | 1170 | 95.1% |
| [30] | ThinPrep Cytologic Test (TCT) images | Mask R-CNN + XGBoost | 400 | 91.92%±5.2  96.21%±4.1 |

Note: This is addressed in subsection (4.3.3) lines 516-540 .

Q7. Are the datasets available for public use? This would really be very helpful to other researchers who may wish to validate their algorithms on this dataset.

We are seeking to make the data fully and publicly available and we are navigating the necessary ethical approvals for releasing the data. Currently all the data for this study will be available on request for non-commercial and academic purposes from the director of iCAIRD ([david.harrison@st-andrews.ac.uk](mailto:david.harrison@st-andrews.ac.uk)).

Note: This is addressed in lines 172-175

Q8. Include the computational requirements for your algorithms; and the anticipated time to save while using the algorithms vs the manual process.

The pathologists that annotated the whole slide images believe, the potential time to examine the tissue and make a diagnosis on a case is approximately five minutes. The diagnosis time of our developed algorithm on the all cases in the test set, running on the NVIDIA Tesla V100 GPU, on average was 1.5 minutes per case. This algorithm runs in advance of a pathologist looking at the slide, and can save pathologist's time to a great extent.

Note: This is addressed in lines 510-515
