# Supplementary material for: Automated reporting of cervical biopsies using artificial intelligence
Source: PLOS Digit Health. 2024 Apr 22;3(4):e0000381. doi: 10.1371/journal.pdig.0000381 (PMC11034655; doi:10.1371/journal.pdig.0000381)
Supplement: Attachment — Submitted filename: PDIG-D-22-00313_Comments - Second_Review.docx [file pdig.0000381.s003.docx]

**First Review**

The paper presents development of an Artificial Intelligence (AI) algorithm for automated diagnosis of cervical cancer from cervical biopsies. The paper presents an interesting topic given the high prevalence of cervical cancer globally; and the need for early and accurate diagnosis. The paper acknowledges that previous work has been done in the field, but mentions some of their limitations including manually extracting features (handcrafted), that need expert domain knowledge and the procedure is laborious and time-consuming. The authors have used a number of recent articles; and the data acquisition, curation, feature selection and classification steps are very clear.

However, the following comments should be addressed to make the manuscript more readable.

Please indicate out of the 2539 whole slide images (WSIs), the number of images that were obtained from Glasgow Royal Infirmary (NG), Southern General Hospital (SG), Royal Alexandria Hospital (RAH) and Queen Elizabeth University Hospital (QEUH).

Please indicate the imaging parameters of the Philips UFS Scanner including resolution, FOV; and the Magnification of the equipment.

On line 140, you mention that two of the labs (labs 6 & 8) and 10% of WSIs from other labs which were randomly. Please indicate which are the labs 6 & 8 mentioned in the manuscript.

Justify why you selected 33% of the training slides as your test train split. Was increasing or reducing this affecting your results in any way (Line 142). However, your Table 1 indicates otherwise. Please clearly explain your test train split.

Avoid putting tables in between a sentence. Place Table 2 after paragraph 297 to improve readability.

XGBoost classifier is an excellent algorithm that has been used by several other authors. Can you please compare your results (probably some metrics of the confusion matrix) with some of the state of art available algorithms with a special focus on the XGBoost classifier variations proposed by different authors.

Are the datasets available for public use? This would really be very helpful to other researchers who may wish to validate their algorithms on this dataset.

Include the computational requirements for your algorithms; and the anticipated time to save while using the algorithms vs the manual process.

**Second Review**

- The authors have really responded to all the comments raised in the first review; and I find the paper fit for publication.
